# Prognostics of unsupported railway sleepers and their severity diagnostics using machine learning

**DOI:** 10.1038/s41598-022-10062-w

**Published:** 2022-04-11

**Authors:** Jessada Sresakoolchai, Sakdirat Kaewunruen

**Affiliations:** University of Birmingham, Birmingham, B15 2TT UK

**Keywords:** Civil engineering, Mechanical engineering

## Abstract

Railway sleepers are safety–critical components of a railway structure. They support ballasted track superstructure and are a critical factor in track geometry and track components’ deterioration. Unsupported sleepers are a common issue incurred after tracks have been utilized. When unsupported sleepers are present, they cause differential settlements of track superstructures, additional dynamic loading, and excessive train-track vibrations which affect passenger comfort, safety, and maintenance cost. This study is the world's first to develop new machine learning models to prognose and better diagnose defect severities of unsupported sleepers aligned with practical track inspection guidelines. Data used to develop machine learning models are based on a verified finite element model with actual field measurements, enabling unbiased full data ranges that govern all defect conditions. Different conditions of unsupported sleepers can be explored by varying locations of unsupported sleepers and the number of unsupported sleepers. Also, other operational parameters can be addressed such as speeds of rolling stock, the roughness of rail surface, and vertical stiffness of wheel-rail contact. In total, 2016 data sets have been obtained. Axle box accelerations are adopted as key indicators for machine learning models. Machine learning techniques used in the study are the convolutional neural network, recurrent neural network, ResNet, and fully convolutional neural network. Data fusion and assimilation have been conducted since the data points are dependent on the train speeds. Our new results reveal a breakthrough essential for real-world applications that the convolutional neural network model provides the best accuracy in both unsupported sleeper prognostics and severity identification. The accuracies of detection and severity identification are 99.34% and 97.02% respectively.

## Introduction

The ballasted track is the most common track superstructure because of its low construction cost, flexible application, low noise and vibration, ease of maintenance, etc. However, the main disadvantage of the ballasted track is the relatively fast deterioration. Its deterioration affects the track geometry which results in lower passenger comfort, safety, and higher maintenance cost. The settlement of the ballast layer starts from voids^1^ which causes excess vibration and high dynamic loading. Because sleepers are fastened with tracks, they will not settle along with the ballast layer. This causes unsupported sleepers. Then, the track geometry will deteriorate even faster because sleepers are not supported by the ballast layers to which the load is transferred from the track.

In the present, unsupported sleepers can be detected using different techniques such as on-board and track-side techniques. Examples of on-board techniques are the use of the Swiss track stiffness measurement vehicle, the rolling stiffness measurement vehicle of the Swedish railway^2^, or other measurement vehicles in other countries such as the USA and China. It can be seen that on-board techniques require special vehicles to perform the inspection which causes high measurement costs. Therefore, the inspection cannot be done frequently. Moreover, on-board techniques have the limitation of low speed and accuracy^1^. Examples of track-side techniques are track deflection technique, cameras, accelerometers^3^, laser array, geophone, and others^4^. The main disadvantage of track-side techniques is the high cost of equipment and sensor installation and software.

One of the techniques that tend to be interested is the use of on-board measurement based on regular rolling stocks. It is cost-efficient, does not interrupt regular service, is fast with the same speed of service operation, and does not require additional installed equipment or sensors. Therefore, the inspection can be done more frequently which results in lower maintenance costs. The most simple and cost-efficient approach is the acceleration measurement. One of the methods that tends to be more popular nowadays is machine learning. From the literature review, the use of machine learning to detect unsupported sleepers has not been comprehensive and seriously studied. The detail will be more explored in “Exploration of sleeper defects and unsupported sleepers detection” section.

This study aims to apply axle box accelerations (ABA) to detect and identify severities of unsupported sleepers using machine learning models. Machine learning techniques used in the study are convolutional neural network (CNN), recurrent neural network (RNN), ResNet, and fully convolutional neural network (FCN). Data for developing machine learning models are generated from a finite element model which the accuracy is verified with the field data. Because outputs’ frequency from finite element simulation is fixed, the number of data points are different depending on the speeds of rolling stock. However, inputs of machine learning models except FCN need to have the same shape or dimension. Data processing is required to reshape inputs. In the study, Fast Fourier transform (FFT) and padding techniques are used to reshape the inputs. To evaluate the performances of machine learning models, accuracies are used.

## Exploration of sleeper defects and unsupported sleepers detection

Due to the increasing load and speed of rolling stock, the track foundation is applied by high impacts under a regular operation. As a result, the deterioration is exponential and leaves the permanent deformation of the track foundation. The settlement tends to occur because of the incident and a gap will form under sleepers. This leads to unsupported sleepers^5^. Augustin et al.^6^ found that mostly over 50% of sleepers were partially unsupported in the reality. Therefore, it is significant to detect unsupported sleepers.

Zhu et al.^5^ studied the dynamic behavior of a track under the unsupported sleeper condition. They applied vehicle–track dynamic interaction theory on their study with a Timoshenko beam concept. In the study, they adjusted the speed and the number of unsupported sleepers of a 1:5 scale model. The speeds were varied between 160 and 320 km/h and the number of unsupported sleepers was varied between 0 and 3. They found that when unsupported sleepers were presented, additional impact loads occurred. This resulted in even more deterioration and spoiled the track quality. Their finds were supported by other studies. Mosayebi et al.^7^ applied the Finite Element Method (FEM) to simulate train-track interaction. They found that unsupported sleepers resulted in additional sleepers’ displacement and impact force. They ranged the speed of rolling stocks between 100 and 200 km/h and the number of unsupported sleepers was ranged from 1 to 2. They tried to find relationships between the peak velocity of a track and the unsupported sleeper stiffness. They found that the displacement of sleepers was more than 90% when a sleeper was unsupported. When two sleepers were unsupported, the displacement increased by more than 190%. The track forces were increased by 1.4 and 1.78 times when one and two sleepers were unsupported respectively. The maximum vibrations were also increased by about 40% when sleepers were unsupported. In addition, they found that the relationships between the peak velocity of a track and the unsupported sleeper stiffness were non-linear. These findings were supported by Zhang et al.^8^, Sadeghi et al.^9^, and Zakeri et al.^10^.

Sleepers are important track components, sleeper defects need to be detected to plan efficient maintenance. Franca and Vassallo^11^ applied image processing to detect and classify types of sleeper defects. For the size of samples, they used 10,116 images that 32,917 sleepers were comprised. The samples consisted of wooden and steel sleepers. They collected data using cameras. The classification accuracies of the developed approach were ranged between 86–93%. Yella et al.^12^ applied SVM to classify the condition of sleepers. They inspected data in Sweden where their samples were wooden sleepers. They stated that manual inspection was commonly used around the world in the railway industry which was slow and expensive. They developed a predictive model to classify the sleeper condition as good or bad (2 classes). The sample sizes were 200 comprising of 144 sleepers in good condition and 56 sleepers in bad conditions. For the predictive model, they applied MATLAB with LNKNET to develop the model. They separated the training and testing data with the proportion of 75% and 25% respectively. As a result, they could achieve the performance of 86% accuracy.

Zheng et al.^13^ applied YOLO V3 which was a popular object detection algorithms to detect sleeper defects. It provided high accuracy and was fast. They collected data which were sleeper images from a metro line. The sample sizes were 1800 images which they separated into 1500 training data and 300 testing data. They used recall as an indicator to evaluate the performance of the model. The precision and recall of the classification were 91.72% and 92.64% respectively. Wang et al.^14^ applied CNN to detect cracks in sleeper images. They proposed to use a two-stage algorithm for detecting sleeper cracks. For the first stage, edge detection was conducted to find crack areas. For the second stage, CNN was used to classify images classified into two classes, sleepers with and without cracks. The accuracy of their model was 95% which was the same as using the CNN model with the raw data. However, using a two-stage algorithm made the training time much faster.

To detect unsupported sleepers, different techniques were applied as mentioned in the introduction. Kim et al.^15^ presented a technique using a portable light falling weight deflectometer (LFWD). This technique was used to measure the gap between sleepers and ballast. Originally, the LFWD was used for highway pavements. An advantage of using the LFWD was it could detect unsupported sleepers immediately. From the study, the authors stated that the LFWD was an easy and rapid technique to detect unsupported sleepers. However, it had some limitations of loads and maximum displacements. They found that the stiffness of unsupported sleepers was only 11.6% of fully supported sleepers. Sysyn et al.^1^ applied numeric simulations to simulate dynamic behavior between track superstructure and rolling stocks. They used time-domain data to detect unsupported sleepers which were displacements. Data collection was done using high-speed video records and digital imaging correlation (DIC). Then, data were statistically analyzed. Another technique that they used to detect unsupported sleepers was machine learning. They processed data using the wavelet scattering feature extraction. Machine learning techniques that they used were support vector machine (SVM) and k-nearest neighbor classifier (KNN). However, they stated that the performance of the developed approach was moderate and could be improved. The maximum accuracies of the SVM and KNN models were 58% and 65% respectively. Therefore, it can be seen that the performance of the machine learning model can be improved by using more advanced machine learning techniques.

It can be seen that although different techniques can be used to detect unsupported sleepers, many techniques required additional equipment or sensor installation which is expensive. Using the machine learning technique is an interesting alternative to develop an unsupported sleeper detection model. In this study, different machine learning models are used to develop models to detect and classify severities of unsupported sleepers. The data used to develop machine learning models are time-series data of ABA because it is convenient and cost-effective to install and practice with regular operation. Moreover, studies about unsupported sleeper detection and severity classification using machine learning models are not popular. Moreover, previous related studies provided moderate performance because the techniques used were not powerful. Therefore, this is a research gap that this study aims to fulfill by using more modern machine learning techniques.

## Development of finite element model

**Figure 1:**
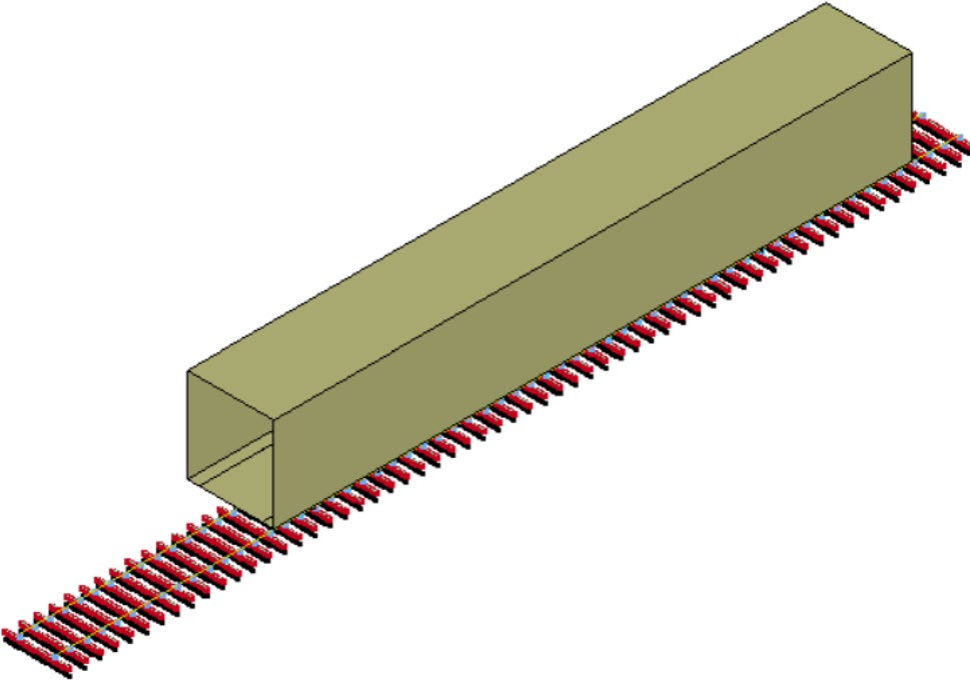
The 3D vehicle-slab FEM model^16^.

This study develops an FEM model to simulate data for machine learning model development. The FEM model is developed based on a study by Li et al.^16^. The FEM model is a 3D vehicle-slab FEM model as shown in Fig. 1. The rolling stock is developed using the multi-body simulation concept. The model is developed using LS-DYNA which is a popular FEM software.

**Figure 2:**
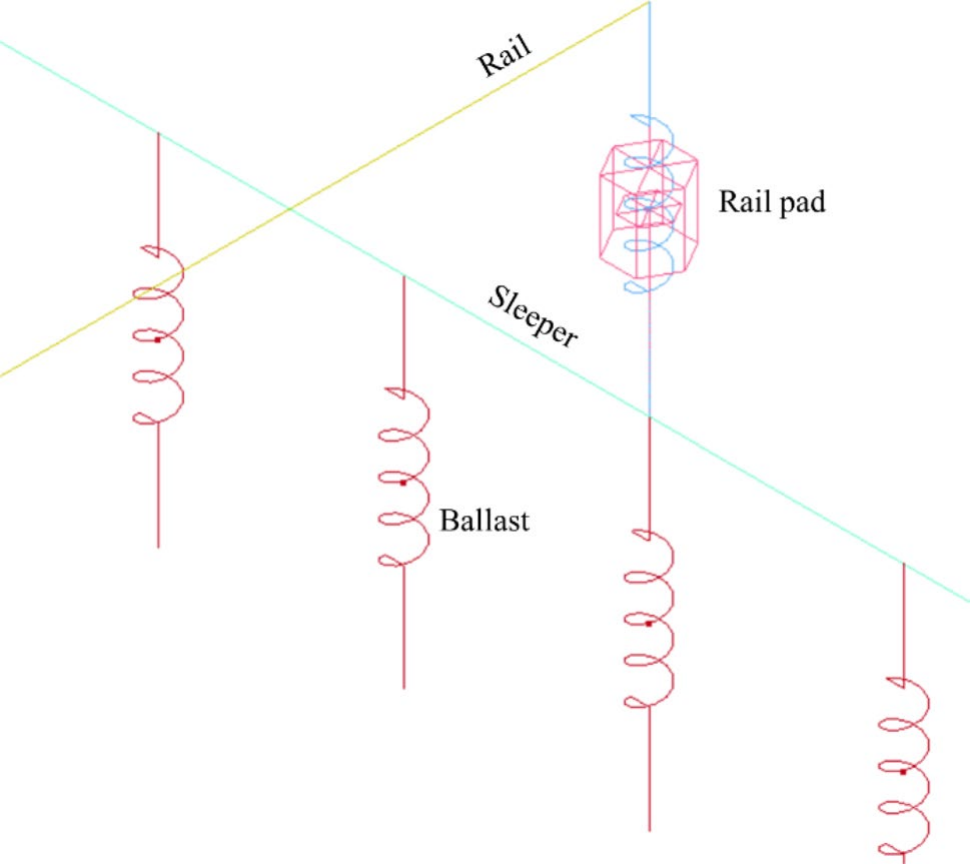
Detailed ballasted track model.

Overall, the rolling stock consists of a car body, two bogies, four wheelsets, primary suspension, and secondary suspension. More detail can be found in the mentioned study. The ballasted track consists of rail, rail pads, sleepers, and ballast. In the model, the rails are Euler beams supported by rail pads which are modeled as springs and dampers. Rail pads are supported by sleepers which are modeled as Euler beams. Then, sleepers transfer loads to ballast which is modeled as spring as shown in Fig. 2. The interaction between wheel and rail is simulated using the built-in keywords in LS-DYNA called *Rail_Track and *Rail_Train using Eq. (1). It is used to calculate wheel-rail contact force where $F$ is the wheel-rail contact force, $K$ is the vertical stiffness of the wheel-rail contact = 1.325 × 10^9^ N/m, ${Z}_{w}$ is the wheel’s vertical displacement, ${Z}_{r}$ is the rail’s vertical displacement, and $\delta$ is the track irregularity.

1 $$F=K\times \left({Z}_{w}-{Z}_{r}-\delta \right)$$

**Figure 3:**
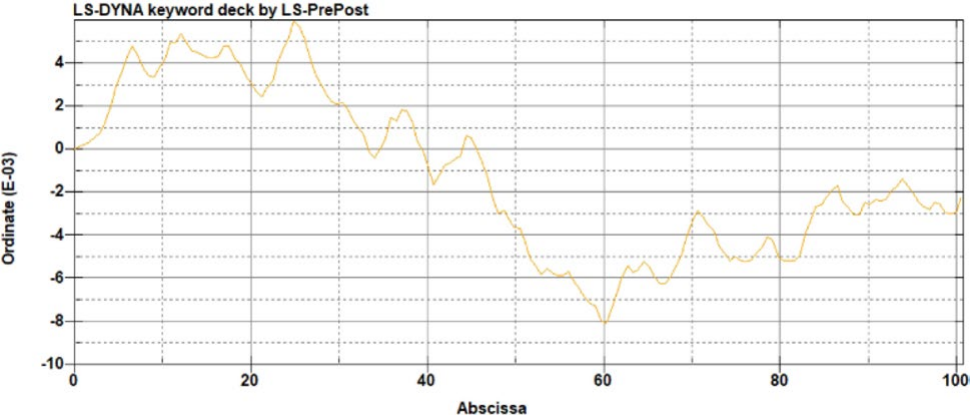
The irregularity of the track.

The irregularity is also included in simulations to reflect the realistic practice as shown in Fig. 3. In simulations, the irregularity is changed between 98 to 101% compared to the original irregularity.

Keywords of S01-SPRING_ELASTIC and S02-DAMPER_VISCOUS are used to simulate the stiffness and damping of rail pads and ballast. To simulate unsupported sleepers, different numbers and locations of springs representing ballast are removed. In the study, the number of unsupported sleepers is ranged between one to four. Speeds of the rolling stock are range from 100 to 200 km/h. The vertical wheel-rail contact stiffness is also varied to reflect the real situation that the track does not have a constant stiffness.

## FEM model verification

To verify the developed model, the developed FEM model is verified with the field tests and previous FEM models. Field data from Cai et al.^17^ is used to verify the model. This section of the track was Sui-Yu Railway. The speed of the rolling stock is 160–220 km/h. To simulate the real operation, the track irregularity is added to the FEM models. Parameters used to verify are wheel-rail contact force and maximum displacements of rail and sleeper. These parameters are referred to the actual field data^17^ and the previous studies^18^ that parameters obtained from the experimental testing and field measurement data. The comparison is shown in Table 1.

**Table 1:** The comparison between verified results and results from the developed FEM model.

Parameters	Field data^18^	This study
Wheel-rail contact force (kN)	100	98.4
Rail displacement at rail seat (mm)	2.606	2.596
Rail displacement at mid span (mm)	2.604	2.415
Sleeper displacement at rail seat (mm)	2.576	2.522
Sleeper displacement at mid span (mm)	2.511	2.352

From the comparison table, the maximum difference is 7% approximately which is less than 10%. Therefore, it can be considered that the results from the developed FEM model are acceptable compared to the experimental results and field measurement data.

## Generation of axle box acceleration data

This study generated numerical data by using simulations of the FEM model as mentioned in “Development of finite element model” and “FEM model verification” sections. The data will be further used to develop machine learning models to detect and evaluate the severity of unsupported sleepers. In the study, ABA is used to train machine learning models. FEM simulations are conducted to generate ABA in different conditions to reflect reality. Moreover, it generalizes data and improves the capability of machine learning models to be practical. For simulations, outputs are recorded with the frequency of 1000 Hz. Data variation can be shown in Table 2. The total length of the section is 35 m. The distance between sleepers is 0.6 m. Therefore, 60 sleepers are supporting this section. To create the data variation, different parameters are varied such as the number of unsupported sleepers, location of unsupported sleepers, speed of the rolling stock, size of irregularity, and vertical stiffness of wheel-rail contact. For the unsupported sleeper condition, every mentioned parameter is varied. For the perfect track condition, the number and location of unsupported sleepers are not varied because they are not available. To imitate the unsupported sleeper condition, springs representing ballast are removed in the FEM model. The original vertical stiffness of wheel-rail contact is 1.325 × 10^9^ N/m. In total, 2016 simulations are run.

**Table 2:** Data variation of FEM simulations.

Parameters	Range
Number of unsupported sleepers	1–4
Location of unsupported sleepers	1–60
Speed of the rolling stock (km/h)	100–200
Size of irregularity (%)	98–101
Vertical stiffness of wheel-rail contact (N/m)	1.06 × 10^9^–1.325 × 10^9^

## Data processing

**Figure 4:**
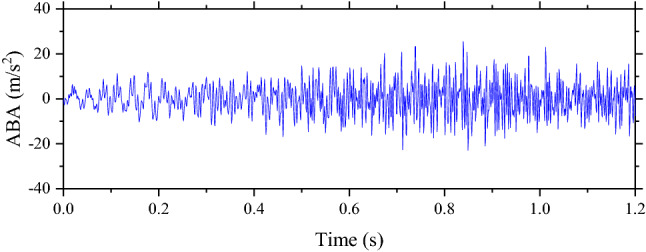
The ABA of the perfect condition when the speed is 100 km/h.

**Figure 5:**
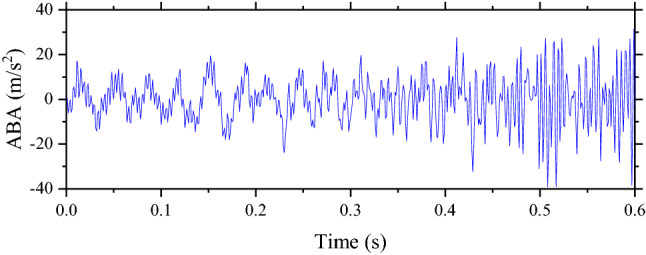
The ABA of the perfect condition when the speed is 200 km/h.

The simulations performed in the study use the output frequency of 1000 Hz. That means any outputs are reported every 0.001 s. Therefore, when the speed of the rolling stock is changed while the length of the track section is the same, the duration of the traveling or simulation time is also changed. When the speed is faster, the number of outputs is decreased. Examples are shown in Figs. 4 and 5 when these two examples present the ABAs of the perfect condition.

From the figures, it can be seen that the simulation time of the simulation with 200 km/h is twice shorter than the simulation with 100 km/h. In this study, machine learning techniques consist of CNN, RNN, ResNet, and FCN. Besides FCN, other models require data or samples with the same shape. Therefore, ABA data need to be processed to make them have the same shape. Two processing techniques are used, namely, padding and FFT. For the padding, it is a basic technique that is used to make samples have the same shape without any change to samples. It can be done by adding 0 s to the samples that the number of input is smaller than others. In this study, the shape of samples with the maximum data point or samples with the lowest speed is fixed based on the frequency of simulations. For other samples with a higher speed, 0 s are added to make them have the same shape as the samples with the lowest speed. For example, if the maximum number of input is 2000, other inputs with the smaller size of data will be added with 0 s to make them have the same shape as the simulations with the maximum size of 2000.

**Figure 6:**
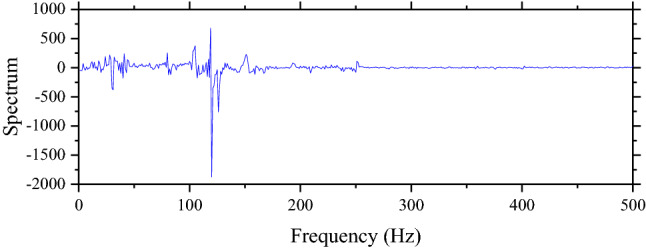
The result from FFT of the ABA of the perfect condition when the speed is 100 km/h.

**Figure 7:**
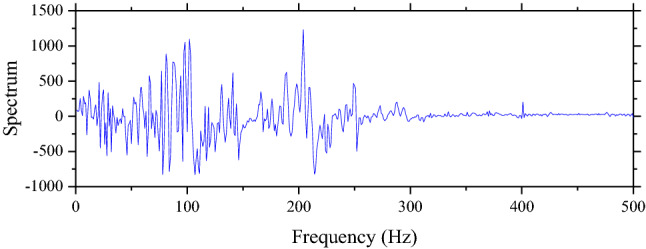
The result from FFT of the ABA of the perfect condition when the speed is 200 km/h.

Another technique that is used to process data is FFT. This technique is based on Fourier Transform that is popular in signaling analysis. This study uses the FFT function in MATLAB to process data. Results of FFT based on Figs. 4 and 5 are shown in Figs. 6 and 7 respectively. After data are processed, they have the same shape and are ready to be fed into machine learning models. In total, the number of samples without unsupported sleepers and 1–4 unsupported sleepers is 612, 360, 354, 348, and 342 respectively.

## Machine learning model development

Data used to develop machine learning models in the study are outputs from FEM simulation which are ABA. Machine learning techniques used to develop models are CNN, RNN, ResNet, and FCN. As mentioned in the data processing part, data need to be processed to make inputs have the same shape. This is the limitation of CNN, RNN, and ResNet because these techniques require the same shape of the input. Therefore, inputs used to develop those three models have to be processed using padding or FFT. However, FCN does not have this limitation due to a benefit of a global pooling layer in the FCN model. Therefore, raw data can be used to be fed into the FCN model. Reasons for choosing these machine learning techniques are they are powerful deep machine learning techniques and can be used to solve varied problems. CNN is popular in pattern recognition which is suitable for problems in this study. RNN is also proven that it is suitable for prediction using time-series data. At the same time, ResNet and FCN are developed based on CNN. ResNet consists of residual blocks comprising convolutional layers and pooling layers. One benefit of ResNet is it contains skip connections that allow data to skip some blocks if the architecture of the model is too complicated unnecessarily. FCN is quite similar to CNN. However, the main difference is it does not contain a dense or fully connected layer. A 1 × 1 convolutional layer is used as the classifier instead of a dense layer. Therefore, data are not flattened. Without dense layers, the model allows data to have variable shapes which are suitable for data in this study.

In the study, two kinds of models are developed to fulfill the objectives of the study. The first is unsupported sleeper detection and the second is unsupported sleeper severity classification. Both kinds of models are for classification. Practically, data will be used to detect unsupported sleepers first. If unsupported sleepers are detected, the data will be further fed into the severity classification model. If not, it can be concluded that the interesting track section does not suffer from unsupported sleepers. For the first step or detection model, it is binary classification. The classes are no unsupported sleepers and unsupported sleepers. For the second step, there are four classes according to the number of unsupported sleepers which range from one to four.

Data are separated into training data and testing data with a proportion of 70/30. Hyperparameter tuning using grid search is conducted to make sure that each model provided the best possible outcome. Hyperparameters that are tuned of each model can be shown in Table 3.

**Table 3:** Hyperparameter tuning of each model.

Models	Hyperparameters	
CNN	Number of convolutional layers Filter Kernel size Number of pooling layers Pool size	Activation function Batch size Optimizer Number of hidden layers Number of hidden nodes
RNN	Number of RNN cells Window size Activation function Batch size	Optimizer Number of hidden layers Number of hidden nodes
ResNet	Number of residual blocks Number of convolutional layers Filter Kernel size Number of pooling layers Pool size	Activation function Batch size Optimizer Number of hidden layers Number of hidden nodes
FCN	Number of convolutional layers Filter Kernel size Number of pooling layers Pool size	Activation function Batch size Optimizer Number of hidden layers Number of hidden nodes

To evaluate the performance of the developed machine learning models, the study uses accuracies as the main criteria. The accuracies can be calculated using Eq. (2) where $TP$ is true positives, $TN$ is true negatives, $FP$ is false positives, and $FN$ is false negatives.

2 $$Accuracy=\frac{TP+TN}{TP+TN+FP+TN}$$

## Experimental results and analysis

### Unsupported sleeper detection

From the machine model development for detecting unsupported sleepers, the accuracy of each model is shown in Table 4.

**Table 4:** accuracies of unsupported sleeper detection.

Models	Accuracies (%)		
	FFT	Padding	Raw data
CNN	99.17	100.00	–
RNN	98.18	80.17	–
ResNet	83.64	93.39	–
FCN	–	–	95.21

**Figure 8:**
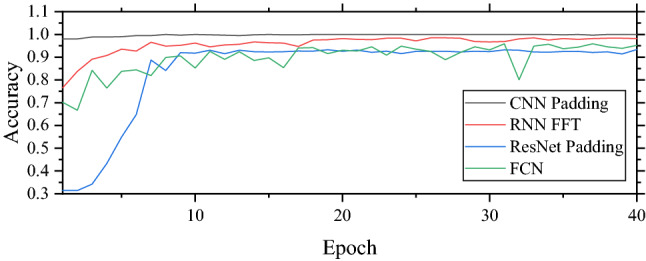
Accuracies of testing data on unsupported sleeper detection.

From the table, it can be seen that each model performs well. The accuracy of each model is higher than 90% when the data processing is appropriate. CNN performs the best based on its accuracies. When CNN is applied with FFT and padding, the accuracies are the first and second highest compared to other models. For RNN and ResNet, the accuracies are higher than 90% when specific data processing is used. However, the accuracies become 80% approximately when another data processing technique is used. For FCN, data processing is not needed. The FCN model can achieve an accuracy of 95%. From the table, the models with the highest accuracy are CNN, RNN, FCN, and ResNet respectively. The complicated architecture of ResNet does not guarantee the highest accuracy. Moreover, the training time of ResNet (46 s/epoch) is the longest followed by RNN (6 s/epoch), FCN (2 s/epoch), and CNN (1 s/epoch) respectively. It can be concluded that the CNN model is the best model to detect supported sleepers in this study because it provides the highest accuracy or 100% while the training time is the lowest. At the same time, easy data processing likes padding is good enough to provide a good result. It is better than FFT in the CNN model which requires longer data processing. The accuracy of testing data of each model is shown in Fig. 8.

The tuned hyperparameters of the CNN model with padding data are shown in Table 5.

**Table 5:** Hyperparameter tuning of CNN model with padding data for detection.

Hyperparameters	Values
Number of convolutional layers	2
Filter	64 (conv1), 32 (conv2)
Kernel size	7
Number of pooling layers	2
Pool size	2
Activation function	ReLu
Batch size	64
Optimizer	Adam
Number of hidden layers	2
Number of hidden nodes	100

Compared to the previous study, Sysyn et al.^1^ applied statistical methods and KNN which provided the best detection accuracy of 65%. The accuracy of the CNN model developed in this study is significantly higher. It can be assumed that the machine learning techniques used in this study are more powerful than the ones used in the previous study. Moreover, CNN is proven that it is suitable for pattern recognition.

### Unsupported sleeper severity classification

For the unsupported sleeper severity classification, the performance of each model is shown in Table 6.

**Table 6:** accuracies of unsupported sleeper severity classification.

Models	Accuracies (%)		
	FFT	Padding	Raw data
CNN	90.28	92.89	–
RNN	71.56	33.89	–
ResNet	51.18	92.42	–
FCN	–	–	81.28

**Figure 9:**
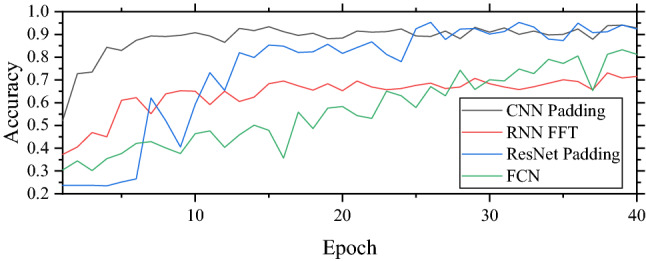
Accuracies of testing data on unsupported sleeper severity classification.

From the table, it can be seen that the CNN model still performs the best with an accuracy of 92.89% and provides good results with both data processing. However, the accuracies of RNN and ResNet significantly drop when unsuitable data processing is conducted. For example, the accuracy of the RNN model with padding drops to 33.89%. The best performance that RNN can achieve is 71.56% which is the lowest compared to other models. This is because of the limitation of RNN that vanishing gradient occurs when time-series data is too long. In this study, the number of data points for padding data is 1181 which can result in the issue. Therefore, RNN does not perform well. ResNet performs well with an accuracy of 92.42% close to CNN while the accuracy of FCN is fairly well. For the training time, CNN is the fastest model with the training time of 1 s/epoch followed by FCN (2 s/epoch), RNN (5 s/epoch), and ResNet (32 s/epoch) respectively. From these, it can be concluded that the CNN model is the best model for unsupported sleeper severity classification in this study. Moreover, it can be concluded that CNN and ResNet are suitable with padding data while RNN is suitable with FFT data. The accuracy of testing data of each model is shown in Fig. 9.

The confusion matrix of the CNN model is shown in Table 7.

**Table 7:** Confusion matric of the CNN model.

	Predicted number of unsupported sleepers			
	1	2	3	4
**True number of unsupported sleepers**				
1	121	1	0	0
2	6	94	6	0
3	1	7	102	3
4	1	1	4	75

To clearly demonstrate the performance of each model, precision and recall are shown in Table 8.

**Table 8:** Precision and recall of each model of severity classification.

Number of unsupported sleepers	1	2	3	4
**CNN padding**				
Precision	0.94	0.91	0.91	0.96
Recall	0.99	0.89	0.9	0.93
**RNN FFT**				
Precision	0.59	0.86	0.75	0.75
Recall	0.92	0.68	0.64	0.64
**ResNet padding**				
Precision	0.98	0.81	0.98	0.96
Recall	1.00	0.96	0.8	0.95
**FCN**				
Precision	0.89	0.81	0.81	0.73
Recall	0.97	0.81	0.73	0.76

From the table, the precisions and recalls of CNN and ResNet are fairly good with values higher than 80% while RNN is the worst. Some precisions of RNN are lower than 60% which cannot be used in realistic situations. CNN seems to be the better model than ResNet because all precisions are higher than 90%. Although some precisions of ResNet are higher than CNN, the precision of class 2 is about 80%. Therefore, the use of the CNN model is better.

For hyperparameter tuning, the tuned hyperparameters of CNN are shown in Table 9.

**Table 9:** Hyperparameter tuning of CNN model with padding data for classification.

Hyperparameters	Values
Number of convolutional layers	2
Filter	64 (conv1), 32 (conv2)
Kernel size	5 (conv1), 7 (conv2)
Number of pooling layers	3
Pool size	3
Activation function	ReLu
Batch size	8
Optimizer	Adam
Number of hidden layers	2
Number of hidden nodes	100 (dense1), 50 (dense2)

## Conclusion

This study applies the machine learning techniques with the FEM model to detect and classify the severity of unsupported sleepers. Numerical data are generated using FEM simulations where FEM models are validated for accuracy by full-scale measurements and field data. This study considers the number of unsupported sleepers equal to one to four. ABA is used as a feature to train machine learning models. CNN, RNN, ResNet, and FCN are used to develop models. Because shapes of input are varied based on the speed of rolling stocks, data processing is required for CNN, RNN, and ResNet because they require input to have the same shape. However, FCN supports the different sizes of input so it does not need processed input. The total number of samples is 2016. From the machine learning model development, CNN is the best model in this study for detecting and classifying the severity of unsupported sleepers. It can achieve an accuracy of more than 90% and its training time is the shortest. It is found that CNN is suitable for padding data which is a simple technique and requires less time than FFT.

The results from this breakthrough discovery can be used in the reality to detect and classify the severity of unsupported sleepers. The developed approach can be easily done using regular rolling stocks which are used for regular operations without costly installation. This study exhibits that using ABA is potentially sufficient to detect unsupported sleepers. Moreover, the approach is much more cost-effective, fast, and does not disturb the regular operation.

A limitation of this study is the input used to develop machine learning models is simulated data from verified FEM models. The rationale is that the verified FEM models can enable unbiased and comprehensive data sets. The use of field data (which could be rather biased) to train machine learning models will however provide better demonstration of the machine learning applications when dealing with operational uncertainties.

To further develop the study, more variety of data can be added such as track properties and rolling stock characteristics. Further field data can be included to support the creditability of the experiment.
